# Loxoscelism: Advances and Challenges in the Design of Antibody Fragments with Therapeutic Potential

**DOI:** 10.3390/toxins12040256

**Published:** 2020-04-16

**Authors:** Sabrina Karim-Silva, Alessandra Becker-Finco, Isabella Gizzi Jiacomini, Fanny Boursin, Arnaud Leroy, Magali Noiray, Juliana de Moura, Nicolas Aubrey, Philippe Billiald, Larissa M. Alvarenga

**Affiliations:** 1 Laboratório de Imunoquímica, Departamento de Patologia Básica, Universidade Federal do Paraná, Curitiba CEP 81531-980 PR, Brazil; sabrinaimtair@yahoo.com.br (S.K.-S.); abeckerfinco@gmail.com (A.B.-F.); bella.jiacomini@gmail.com (I.G.J.); julianademoura@ufpr.br (J.d.M.); 2 INRA, UMR 1282, Faculté de Pharmacie, Université de Tours, 37200 Tours, France; fanny.boursin@univ-tours.fr (F.B.); nicolas.aubrey@univ-tours.fr (N.A.); 3 IPSIT (Chiral-ist), School of Pharmacy, University Paris-Saclay, 92296 Châtenay-Malabry, France; leroy.arnau@wanadoo.fr (A.L.); magali.noiray@universite-paris-saclay.fr (M.N.); 4 MCAM, UMR MNHN-CNRS 7245, Muséum national d’Histoire naturelle, 75231 Paris, France

**Keywords:** venom, antivenom, neutralization, loxosceles, sphingomyelinase D, humanization, scFv

## Abstract

Envenoming due to *Loxosceles* spider bites still remains a neglected disease of particular medical concern in the Americas. To date, there is no consensus for the treatment of envenomed patients, yet horse polyclonal antivenoms are usually infused to patients with identified severe medical conditions. It is widely known that venom proteins in the 30–35 kDa range with sphingomyelinase D (SMasesD) activity, reproduce most of the toxic effects observed in loxoscelism. Hence, we believe that monoclonal antibody fragments targeting such toxins might pose an alternative safe and effective treatment. In the present study, starting from the monoclonal antibody LimAb7, previously shown to target SMasesD from the venom of *L. intermedia* and neutralize its dermonecrotic activity, we designed humanized antibody V-domains, then produced and purified as recombinant single-chain antibody fragments (scFvs). These molecules were characterized in terms of humanness, structural stability, antigen-binding activity, and venom-neutralizing potential. Throughout this process, we identified some blocking points that can impact the Abs antigen-binding activity and neutralizing capacity. In silico analysis of the antigen/antibody amino acid interactions also contributed to a better understanding of the antibody’s neutralization mechanism and led to reformatting the humanized antibody fragment which, ultimately, recovered the functional characteristics for efficient in vitro venom neutralization.

## 1. Introduction

To date the *Loxosceles* genus is comprised of 139 described spider species, differentially distributed and found in all five continents where different species have been reported [1]. In Brazil, *L. intermedia, L. gaucho*, and *L. laeta* are of particular medical concern, as in 2019 the number of reported envenomations was 8490, of which 11 were fatal for humans [2,3]. The diagnosis for loxoscelism is often impaired and belatedly given as bites are usually painless and often go unnoticed, including clinical manifestations that appear only several hours afterwards. Symptoms start two to eight hours post-event and are marked by an intense inflammatory reaction at the bite site, followed by local necrosis that can lead to ulcers of variable sizes. Such lesions often heal within 6 to 8 weeks, but can leave lasting scars which may even require surgical excision [4]. Viscerocutaneous, also designated as systemic loxoscelism, is the most serious clinical manifestation and accounts for up to 27% of cases [5]. It is characterized by fever, nausea, hematuria, hemoglobinuria, and disseminated intravascular coagulation. Occasionally, extensive hemolysis may lead to acute kidney injury and renal failure, the primary cause of loxoscelism-associated deaths.

Usually, bites result in the intradermal injection of few microliters of venom corresponding to around 50 micrograms of protein. As indicated by 2D electrophoresis, the protein content of the venom has great interspecies similarity, with proteins ranging from 2 to 94 kDa. This includes serine proteases, serine protease inhibitors, hyaluronidases, inhibitor cystine knot (ICK) peptides and phospholipases D (PLD), the latter being the most studied and well-characterized venom components due to their ability to induce dermonecrotic lesions and hemolysis [6]. More than 25 spots immunologically related to PLD toxins have been identified in the *L. intermedia* venom, most of them being SMase D-related [7]. A great number of studies has been carried out on these toxins. Nine isoforms of PLDs have been recombinantly produced and expressed as soluble and active enzymes that reproduce most of the toxic effects observed in loxoscelism [8,9,10,11,12]. X-ray crystallography analysis of recombinant LiRecDT1 SMase D (SMase D LiRecDT1) from *L. intermedia*, both wild-type and H12A-mutant forms, are available and the catalytic pocket of the enzyme is well-identified [13]. This and other reports reveal important insights into the enzymatic properties of each one of the isoforms, but also underline clear differences in the hydrolytic ability of PLD isoforms within the *Loxosceles* genus.

To date, there is no consensus treatment for the management of patients who are admitted to the hospital 12 to 24 h after the bite [14,15]. Symptomatic and non-specific treatments have been implemented in most countries for the treatment of the less critical cases. In South America, horse polyclonal antivenoms are available and usually infused intravenously to all patients showing viscerocutaneous loxoscelism. These preparations are mostly comprised of F(ab)’_2_, but also whole IgGs (Peru) [14,15,16]. They target all the components of the venom and may neutralize toxins by various mechanisms, including direct inhibition of the toxin’s catalytic site, steric hindrance, and allosteric inhibition. However, these *Loxosceles* antivenoms have often been less successful than those produced for the treatment of snake envenomings, in light of their effectiveness [15]. There is no direct relationship between toxicity of the venom’s molecules and their immunogenicity, and antibodies raised against *Loxosceles* dermonecrotic toxins often show low interspecies cross-reactivity [17,18]. In addition, such conventional antivenoms based on animal immunization belong to the category of blood-based products as defined by the regulatory authorities, with safety concerns, not chemically well-defined components, and high batch to batch variability. Polyclonal antibodies present limited specific activity, thus they might bind to toxin components, but not necessarily neutralize them and could be effectively substituted with specific anti-SMase D antibodies [19]. In this context, recombinant binding-proteins with toxin neutralization potential could be an interesting alternative.

One promising and suitable strategy for whole venom neutralization consists in using monoclonal or oligoclonal antibody fragments. Several antibodies discovered against *Loxosceles* venom components have been previously generated [17,20,21]. However, only one (LimAb7) satisfies all the criteria required in terms of specificity, affinity, and neutralizing capacity [20]. LimAb7 specifically binds to 32–35 kDa components of *L. intermedia* venom and is reactive to the SMase D LiRecDT1 toxin. This antibody was also shown to neutralize the venom’s dermonecrotic activity in rabbits, while recombinant antibody fragments preserved antigen-binding affinity and the ability to neutralize the venom in vitro [22]. Nonetheless, the potential immunogenicity of antibodies from mouse origin is a major barrier to infusion in humans. This drawback can be overcome by modifications on the framework regions of the antibody’s V-domains, through a process termed antibody humanization, which reduces its immunogenic potential while maintaining the bioactivity of the antibody molecule [23,24]. All things considered, we hereby designed and evaluated a recombinant humanized antibody fragment constructed from LimAb7 complementarity determining regions (CDRs). During this process, we clearly identified some blocking points that could impact in the antibody’s antigen-binding activity, neutralizing capacity, and pharmaceutical development. In silico mapping of the antigen/antibody interaction also contributed to a better understanding of its intrinsic neutralization mechanism and has led us to reformat the humanized antibody fragment which, ultimately, recovered the functional characteristics required for efficient in vitro neutralization of the venom.

## 2. Results

### 2.1. Design of a Humanized scFv Anti- L. Intermedia Venom

**Figure 1:**
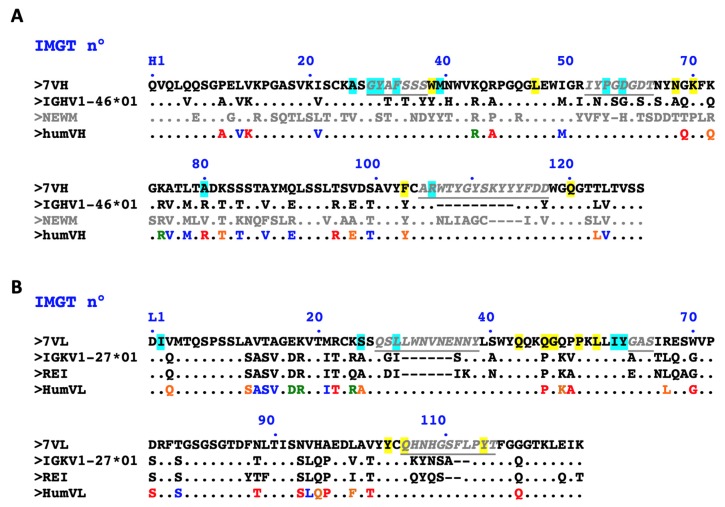
Design of humanized LimAb7 V-domains. (**A**) Sequence alignment of the mouse LimAb7 IGHV (7VH) with human germline sequence IGHV1-46*01, the NEWM protein sequence (“fixed framework” strategy) and the humanized LimAb7 IGHV (humVH) that retains antigen-binding activity. (**B**) Sequence alignment of the mouse LimAb7 IGKV (7VL) with human germline sequence IGKV1-27*01, the REI protein sequence (“fixed framework” strategy) and the humanized LimAb7 IGKV (humVL) that retains antigen-binding activity. CDRs according to IMGT are in italic, underlined, grey. Residues at key sites for canonical structures are highlighted in light blue. Residues buried in VH/VL interfaces are highlighted in yellow. Based on the physico-chemical classes of the amino acids (AA), differences in the framework regions of mouse LimAb7 and its humanized variants are classified into very similar AA (green), similar AA (blue), dissimilar AA (orange) and very dissimilar AA (red).

Structural analysis of LimAb7 allowed us to identify all three CDRs of each V-domain (Figure 1). Length-independent canonical class and sub-class for the non-H3 CDRs were identified as L1–similar to 3/17A; L2–1/7A; L3–similar to 5/11A; H1– similar to 1/10A; H2–2/10A. The packing angle of VH and VL domains, influential to the topography of the antigen-combining site, was predicted to be −42.8 [25]. The humanness score of the V-domain sequences (−1.132 and −1.164 for VH and VL, respectively) clearly indicated a high risk of immunogenicity, all parameters being accounted for further humanization [26].

Sequence database searches allowed us to identify human germline genes closely related to LimAb7 V-domains (IGHV and IGKV) and perform an alignment of the deduced AA residues sequences. We also aligned the LimAb 7 sequences with NEWM (PDB: 7FAB) and REI (PDB: 1REI) myeloma antibody sequences for VH and VL, respectively. These sequences are often used in a “fixed framework” strategy for antibody humanization [24]. However, NEWM was dropped of this study due to its substantial sequence dissimilarity with IGHV-LimAb7 (<50% identity), whilst the human germline IGKV sequence (IGKV1-27*01) was identified as one of the most similar human template for the kappa chain (60.0% identity).

**Figure 2:**
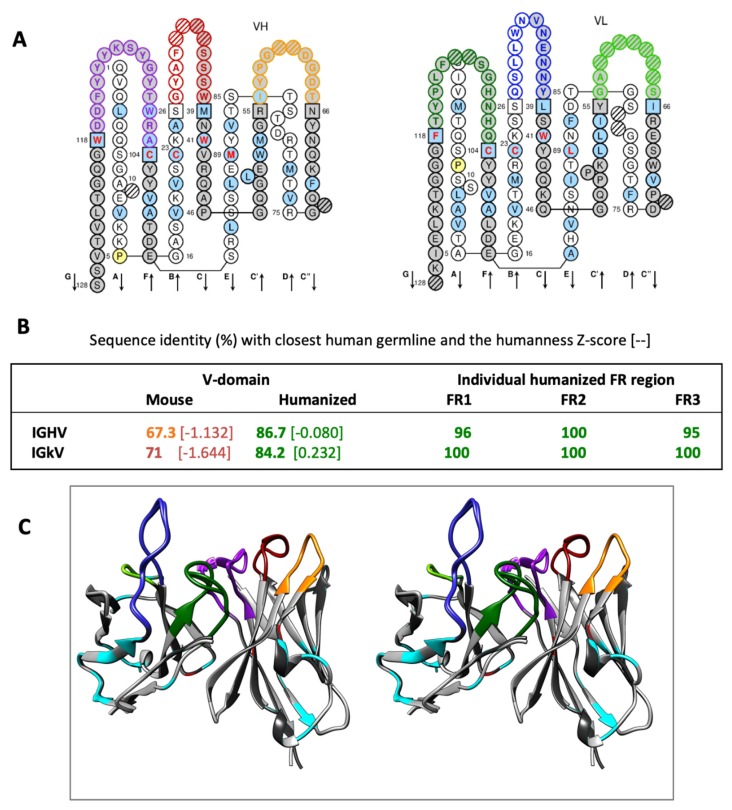
LimAb7 humanized V-domains. (**A**) Secondary structure representation of the variable region sequences in the “collier de perles” format with coloured CDRs (H1: red, H2: orange; H3: purple; L1: blue; L2: light blue; L3: forest green). Arrows indicate β-strands. (**B**) Comparative analysis between the LimAb7 antibody mouse variable region sequences and the modified sequences after humanization with human germline sequences. Percentage of sequence identity with the closest human germline considering the whole V-domain sequence or each individual humanized FR. The humanness Z-score of each domain is also indicated into brackets. (**C**) Stereo view of scFv_15_hLi7. Mutated residues exposed at the surface are in cyan. Residues buried in VH/VL interfaces and mutated upon humanization are in red.

For each of the LimAb7 V-domains, 29 AA residues differed from the human acceptor frameworks (FR). We paid close attention to maintaining cohesion between most residues at positions in VL and VH buried in the interface between the domains. In order to create and secure a PpL binding site that is highly dependent on several residues belonging to IGKV FR1 and residues L90 and L127 as well, the whole FR1 of REI protein was introduced while maintaining residues T (L90) and K (L127) [27]. All throughout the process, the modifications were approved after detailed analysis in order to confirm the ongoing improvement of the humanness Z-score and the decrease of the residual immunogenicity (Figure 2). Canonical class and sub-class CDRs were preserved for L1, L2, L3 and H1 while high similarity with canonical class 2/10A was maintained for H2 (residue H80: preferentially Arg but also Val). The expected VH/VL packing angle was slightly modified upon the process (−42.8 for LimAb7 and −45.4 for the humanized version) but this change was considered acceptable. Lastly, 23 and 26 residues were mutated for IGHV and IGKV, respectively. A 3D structural model of the humanized V-domains of LimAb7 is shown in Figure 2C.

### 2.2. Primary Screening of scFv_15_hLi7

**Figure 3:**
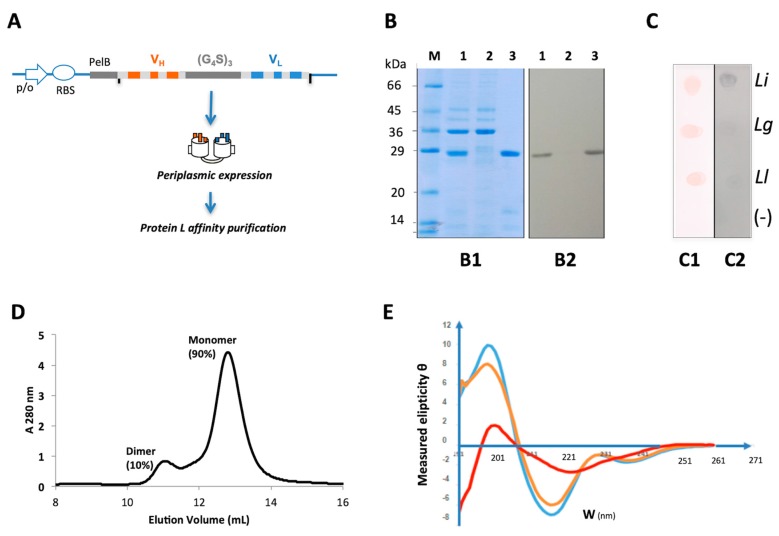
Design, expression and purification of scFv_15_hLi7. (**A**) Schematic representation of the design and expression cassette. The open reading frame is under the control of a T7 promoter and contains a PelB signal sequence for periplasmic expression followed by a cDNA encoding humanized VH and VL fused together via a (Gly_4_Ser)_3_ linker. This allows expression of monomeric untagged scFv_15_hLi7. (**B**) Protein expression scFv_15_hLi7 analysis in periplasmic extract by polyacrylamide gel electrophoresis (SDS-PAGE) and Western blot, before and after affinity purification. **B1,** 12.5% SDS-PAGE gel under non reducing conditions stained with Coomassie blue. The numbers correspond to PpL affinity chromatography column fractions (1) crude periplasmic extract; (2) column flow-through fraction, containing many of the bacterial proteins observed in the crude periplasmic extract; (3) proteins eluted by an acid solution pH 2.8. (M) Molecular weight marker (Sigma M3913). **B2,** nitrocellulose membrane for Western blot analysis developed with PpL-peroxidase conjugate. (**C**) Dot blot analysis of the periplasmic extract containing recombinant scFv_15_hLi7. *L. intermedia (Li), L. gaucho (Lg)*, and *L. laeta (Ll)* venoms (2.5 μg) spotted onto nitrocellulose membrane. **C1**, Confirmation of venom’s presence in the membrane by Ponceau reversible staining. **C2**, nitrocellulose membrane incubated with periplasmic extract containing recombinant scFv15hLi7, developed with PpL-peroxidase. (**D**) Size-exclusion chromatography of the PpL-purified scFv15hLi7 using a calibrated Superdex 75 10/300GL column. (**E**) Far UV-CD analysis of the PpL-purified scFv15hLi7 at 20 °C (blue), 40 °C (orange) and 50 °C (red).

A synthetic codon-optimized bacterial gene encoding the monomeric single-chain antibody fragment scFv_15_hLi7 made up from the humanized VH domain of LimAb7 fused to the humanized VL domain via a flexible 15 residues linker was designed (Figure 3A). Pilot expression was carried in the periplasm of bacteria and the recombinant antibody fragment was isolated from other periplasmic proteins by affinity chromatography using PpL-affinity resin (Figure 3B). No precipitation was observed in the elution peak after neutralization and buffer exchange to PBS pH 7.2. A samples’ electrophoretic profile analysis confirmed the identity of the purified protein which appeared as a unique 29 kDa protein after Coomassie Blue staining, as well as after Western blotting using the PpL-peroxidase conjugate under non-reducing conditions. Dot blot allowed a rapid screening to validate the ability of scFv_15_hLi7 to bind *L. intermedia* venom in a specific manner with no detectable cross-reactivity with *L. laeta* and *L. gaucho* venoms (Figure 3C).

### 2.3. Physico-Chemical Characterization and Stability Analysis of scFv_15_hLi7

Based on the observations stated above, PpL-purified scFv_15_hLi7 was produced at a larger scale at Genscript (Piscataway, NJ, USA) and used for further characterization. SDS-PAGE and Western blotting confirmed correct production and PpL-purification of the recombinant protein. The UV-Vis spectra of the PpL-purified scFv_15_hLi7 was monitored to detect the presence of submicron-sized aggregates. The shape of the spectra was in conformity with that of a soluble protein and confirmed that no significant aggregation/precipitation phenomenon occurred. The value recorded at 320 nm which reflects the scattering of light by large aggregates present in the sample was very low as compared to the value recorded at 280 nm, the ratio A_320nm_/A_280nm_ being 2.5%.

PpL-purified scFv_15_hLi7 was analysed after SE-HPLC in order to confirm purity and for the detection of nanoaggregates and/or degradation products of the scFv in solution (Figure 3D). Two peaks of elution were observed. The smaller peak eluted at a volume ~11 mL corresponded to proteins exhibiting an apparent Mr of 40–50 kDa whereas the major peak, accounting for 90% of the total protein amount, eluted at ~13 mL, corresponding to proteins with an apparent Mr of 25–30 kDa. Based on that apparent Mr, the first peak was expected to be a dimeric form of the scFv related to either a misfolding occurring during the expression or an aggregation phenomenon during the purification process, such as elution in acidic condition. The second peak was likely to be the monomeric form of scFv_15_hLi7.

We also used circular dichroism spectroscopy as a tool to monitor structural stability of scFv_15_hLi7 (Figure 3E). We did not observe any major changes at temperatures beneath 35 °C. At 40 °C, a decrease of the ellipticity at 200 nm was observed and the ellipticity continued to decrease at 50 °C and 60 °C with a concomitant increase of the ellipticity at 218 nm, in the same range of temperature. The shape of the CD spectrum of scFv_15_hLi7 solutions at temperature under 35 °C showed features of a β-sheet-enriched structure, with a minimum at 218 nm and a maximum at ~ 201 nm, typical of antibody-like structures. As temperature increases above 35 °C, the CD spectra of scFv_15_hLi7 displayed no isodichroic point. As a result, the folding of scFv_15_hLi7 cannot be described by a two-state equilibrium, revealing the multidomain nature of the scFv_15_hLi7 fragment. At temperatures above 35 °C, the protein structure of scFv_15_hLi7 is unstable and likely unfolded. Other than CD spectroscopy, we carried out a nano-differential scanning fluorimetry (nano-DSF) analysis. In using a 10 μM solution of PpL-purified scFv_15_hLi7, we observed a melting temperature (T_M_) of 42.2 °C with an onset temperature of denaturation of 37.0 °C and an onset temperature of aggregation of 41.8 °C.

### 2.4. scFv_15_hLi7 Antigen-Binding Characterization

**Figure 4:**
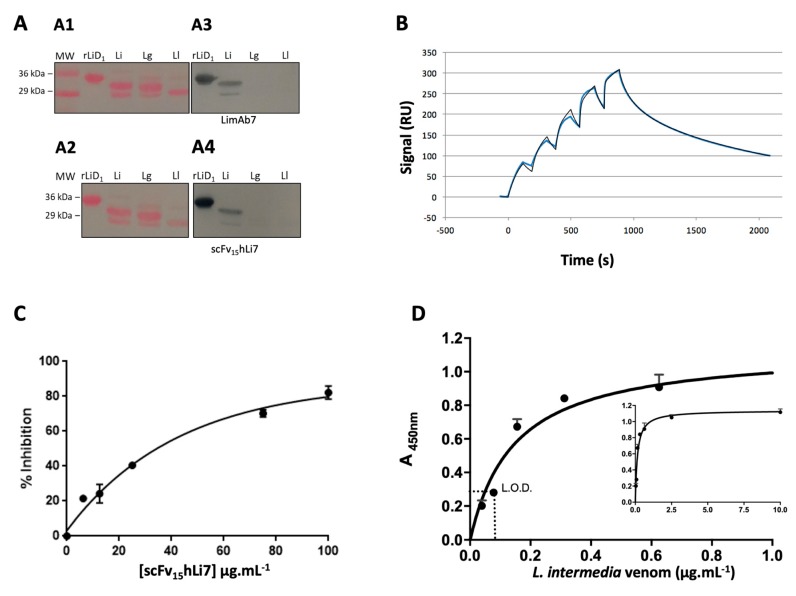
Functional characterization of PpL-purified scFv_15_hLi7. (**A**) Western blot analysis of the LimAb7 and periplasmic extract containing recombinant scFv_15_hLi7. *L. intermedia (Li), L. laeta (Ll) and L. gaucho* (Lg) (10 μg of each venom) or SMase D LiD1 (rLiD1) (10 μg) were resolved in a 15% polyacrylamide gel by SDS-PAGE under non-reducing conditions and transferred to a nitrocellulose membrane **A1**–**A2**, Confirmation of venom’s presence in the membrane by Ponceau reversible staining. **A3**, membrane incubated with LimAb7 (20 µg·mL^−1^) and developed with peroxidase-conjugated rabbit anti-mouse IgG. **A4,** membrane incubated with periplasmic extract containing recombinant scFvLi_15_hLi7 and developed with peroxidase-conjugated PpL. (**B**) Interaction of immobilized rLiD1 (black line) with increasing concentration of PpL-purified scFv_15_hLi7 (0.125; 0.25; 0.5; 1.0; 2.0 μM) analyzed by surface plasmon resonance (SPR) (Biacore 100 and fitting (blue line) with heterogeneous analyte model, monomer 90%, dimer 10%). (**C**) Competitive ELISA: immobilized *L. intermedia* (10 µg·mL^−1^) and added 1 µg·mL^−1^ of LimAb7 with increasing amounts of scFv_15_hLi7. Immunocomplexes were revealed using peroxidase-conjugated anti-mouse Fc antibodies. (**D**) Sandwich ELISA: immobilized horse anti-*L. intermedia* venom F(ab)’_2_ (10 µg·mL^−1^) and captured *L. intermedia* venom gradient. 20 µg·mL^−1^ of scFv_15_hLi7 were added. Immunocomplexes were revealed using peroxidase-conjugated PpL. The inserted graph is the same graph with smaller x-axis scale.

The recognition profile and specificity of the scFv_15_hLi7 against *Loxosceles* venoms and recombinant SMase D LiD1 toxin was identified after the electrophoretic migration of the venom components followed by a nitrocellulose membrane transfer of these proteins and evaluation of the antibody-fragment binding capacity as compared to the parental mouse IgG. When scFv_15_hLi7 was assessed against the venom of different *Loxosceles* species, only the *L. intermedia* venom led to a recognition profile of two bands in the 32–35 kDa molar range, suggesting that the epitope recognized by this fragment is shared by several SMase D isoforms only pertaining to *L. intermedia* (Figure 4A).

Binding affinity of PpL-purified scFv_15_hLi7 and kinetics for binding to immobilized LiD1 were analyzed in real time (Figure 4B). According to SE-HPLC analysis, the heterogeneous analyte model, which consists of two populations of molecules capable to bind to the immobilized target independently, was used. The kinetic constants measured for the monomeric scFv_15_hLi7 representing 90% of the antibody population were k_a_ = 18.2 × 10^4^ M^−1^ s^−1^, k_d_ = 103 × 10^−4^ s^−1^ resulting in a dissociation constant K_D_ = 56.6 nM. Under similar experimental conditions, mouse dimeric scFv_5_Li7 and IgG LimAb7 were previously shown to have significant better kinetic characteristics with a K_D_ of 0.98 nM and 0.085 nM, respectively [22].

The competitive ELISA assay also allowed us to evidence the ability of scFv_15_hLi7 to compete with the parental IgG LimAb7 in a dose-dependent fashion. Figure 4C shows the inhibition profile in the presence of the recombinant antibody fragment. Higher concentrations of the fragment have shown to inhibit the binding of the parental IgG to the venom components and thereby result in a decrease in reactivity. It was estimated that about 38 μg·mL^−1^ of scFv_15_hLi7 is required to inhibit 50% of LimAb7 binding. A sandwich ELISA confirmed the ability of scFv_15_hLi7 to bind to toxins of the *L. intermedia* venom in a dose-dependent and saturable manner with a lower limit of detection of up to 78 ng·mL^−1^ of soluble toxin (Figure 4D). Following this functional characterization, we moved to the evaluation of scFv_15_hLi7′s ability to neutralize the hemolytic activity of *L. intermedia* venom. Unfortunately, we couldn’t observe any neutralization, whatever the experimental conditions were (data not shown). This result led us to reconsider the design of the humanized antibody fragment.

### 2.5. Modelling and Docking of LimAb7, scFv_15_hLi7 with LiRecDT1

**Figure 5:**
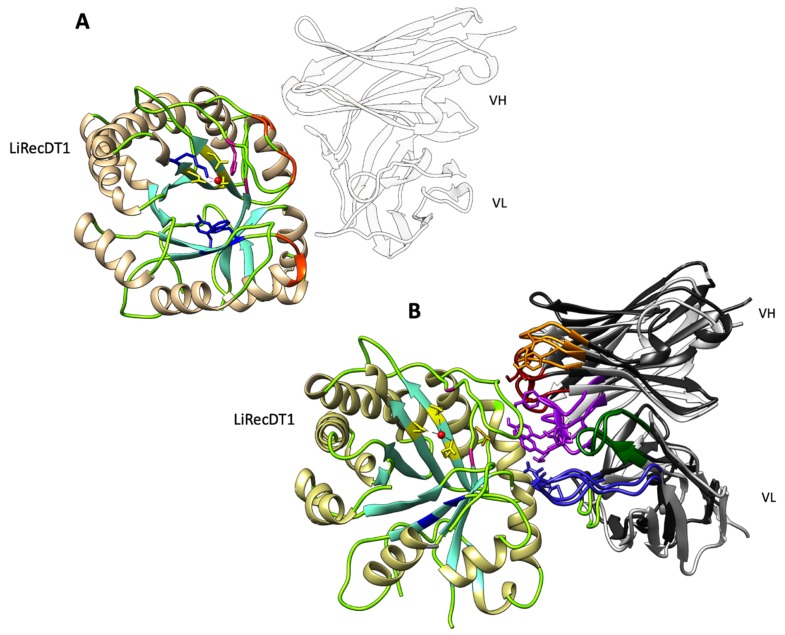
Docking of LimAb7, scFv_15_hLi7 with LidRecDT1. (**A**) Docking results showing antibodies binding sites on the surface of SMase D (LiRecDT1, PDB: 3RLH). Ribbon representations of antibody-Lid1 interactions where the residues proposed to be involved in catalysis (H12A, H47A) are coloured in magenta, magnesium-ion binding (E32A, D34A, D91A) in yellow and substrate recognition (K93A, Y228A, and W230A) are coloured in blue; the amino acid compounds in the predicted epitope for Limab7 are represented in orange. (**B**) Superimposition of the best scoring docking models for LimAb7 and scFv_15_hLi7. The VL and VH CDRs are coloured according to the IMGT colour scheme and the framework regions in black for LimAb7 and grey for scFv_15_hLi7.

In order to better understand LimAb7′s neutralization mechanism, we proceeded to the modeling of LimAb7 and scFv_15_hLi7 V-domains structures prior to in silico docking prediction with putative target SMase D LiRecDT1 (PDB: 3RLH). The molecular docking between LimAb7, scFv_15_hLi7 and their molecular target (PDB: 3RLH) was then performed and the lowest energy score interactions between the antibodies VH/VL and LiRecDT1 are represented in Figure 5. The docking analysis shows LimAb7 VH and VL CDR interactions in accordance with (D233, K234, R235, Y253). Contacts between LimAb7, scFv_15_hLi7 and regions of SMase D LiRecDT1 (K58, K59) (D21, E22, D25), respectively, that have been indicated as highly immunogenic and targets of neutralizing anti-LiRecDT1 antibodies, residues 58–72 (CYGSKKYENFNDFLKGLR) and residues 25–51 (NLGANSIETDVSFDDNANPEYTYHGIP) were also identified. Moreover, contacts between LimAb7 and amino acids G54 and R55 were detected, some of the residues that have been previously indicated as being important for the stabilization of the catalytic loop and substrate interaction are conserved between SMase D LiRecDT1 and SMase LiDI [28,29]. The docking analysis for the scFv_15_hLi7/SMase D LiRecDT1 still shows contacts in the same regions as the murine antibody (LimAb7), however some contacts with substrate-binding relevant amino acids are lost (D233, G54). Furthermore, new contacts have been observed including residues D21, E22, N25, D255 suggesting a displacement in the site of interaction when compared to LimAb7 (Table S1).

### 2.6. Re-Design of Humanized scFv anti- L. Intermedia Venom

**Figure 6:**
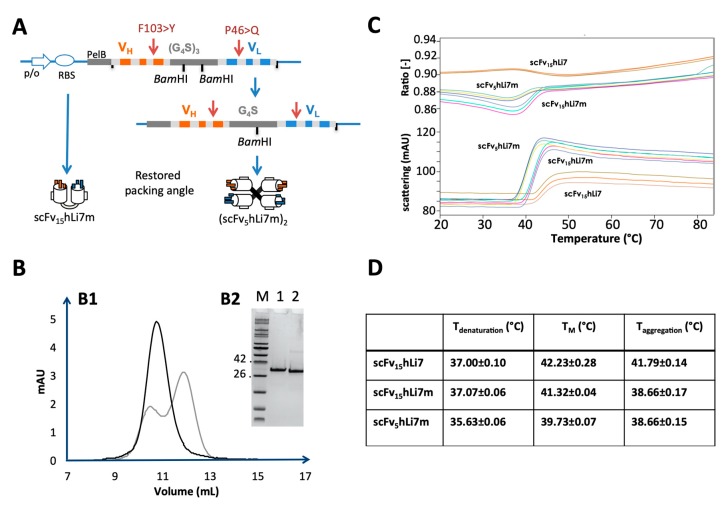
Expression, purification and physico-chemical characterization of scFvhLi7m. (**A**) Schematic representation of the design and expression cassette. The open reading frame contains a PelB signal sequence for periplasmic expression. cDNA encoding humanized VH and VL are fused together via a sequence encoding a (Gly_4_Ser)_3_ linker and cloned in frame with the PelB sequence. Red arrows indicate back mutations. For expression of (scFv_5_hLi7m)_2_, the plasmid was double digested with *Bam*HI and then self ligated. (**B**) Size-exclusion chromatography of the PpL-purified scFv_15_hLi7m (grey) and scFv_5_hLi7m (black) using a calibrated Superdex 75 10/300GL column (**B1**). The insert (**B2**) shows SDS-PAGE analysis of PpL purified scFv_15_hLi7m (1) and scFv_5_hLi7m (2) under reducing conditions. M: Molecular weight marker (kDa). (**C**) Nano-DSF and thermal stability analysis of PpL purified scFv_15_hLi7m, scFv_5_hLi7m in comparison to the first generation scFv_15_hLi7 (each sample was treated in triplicate). (**D**) On set temperature of denaturation, T_M_ and onset temperature of aggregation are indicated in the table.

Considering that the catalytic pocket of SMase D LiRecDT1 and LimAb7 epitope do not overlap according to docking analysis, and also that the humanization process slightly altered the antigen-antibody interaction, we decided to re-design the humanized antibody fragment as follows. First, we introduced two back mutations (F 103 > Y in IGHV and P 46 > Q in IGKV according to IMGT numbering) in the sequence of the humanized antibody fragment. We anticipated that these back mutations should restore the original VH/VL packing angle (−42.8), the original topography of the antigen-binding site and possibly the structural stability of the paratope. Secondly, we produced this new version of humanized antibody in two formats: a monomeric scFv also designated scFv_15_hLi7m (25 kDa) and a larger dimeric scFv_5_hLi7m (50 kDa) (Figure 6). For both recombinant proteins, the yield of production after PpL capture from the periplasm of induced HB2151 bacteria was higher than 1 mg·L^−1^ of culture under standard conditions of culture and induction (2.4 mg·L^−1^ and 1.2 mg·L^−1^ for scFv_15_hLi7m and scFv_5_hLi7m, respectively). In both cases, we did not observe any tendency to aggregation, precipitation or proteolyis as indicated by the low A_320nm_/A_280nm_ ratio (<2.5%) and SDS-PAGE analysis (Figure 6B). Size-exclusion chromatography elution profile indicated that 100 per cent of the scFv_5_hLi7m was produced as a dimer. The scFv_15_hLi7m was mainly produced as a monomeric molecule but we also observed an additional peak corresponding to misfolded proteins or dimeric structures. The nano-DSF analysis performed with both recombinant proteins in comparison with the first generation molecule did not show any improvement of the thermal stability upon the double mutation and dimerization into a diabody molecule (Figure 6C).

**Figure 7:**
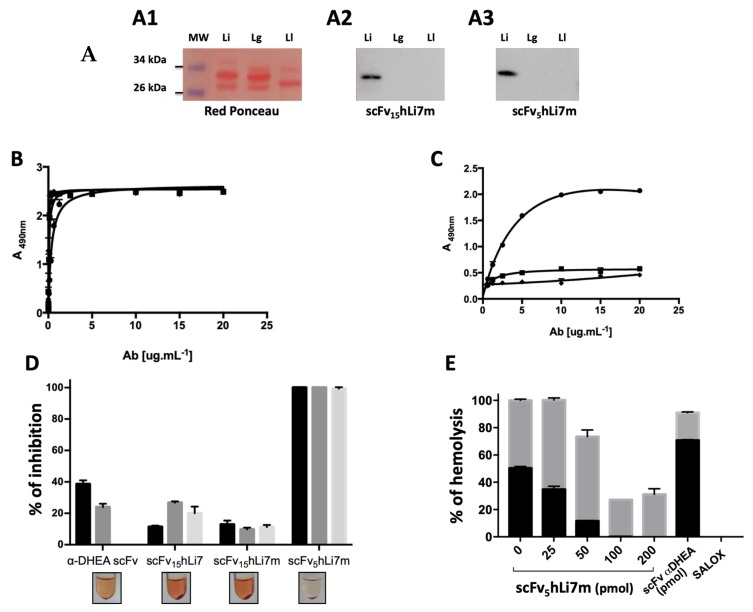
Functional characterization of scFvhLi7m. (**A**) Western blotting after SDS-PAGE 15% of 10µg *Loxosceles* venoms under non-reducing conditions stained with Red Ponceau (**A1)** or incubated with periplasmic extracts of the scFv_15_hLi7m **(A2)** and scFv_5_hLi7m **(A3),** and then developed with peroxidase-conjugated PpL. Li: *L. intermedia*; Lg: *L. gaucho*; Ll: *L. laeta*. (MW) Molecular weight marker (ThermoScientific 26634). (**B**) Indirect ELISA: immobilized SmaseD LiD1 and added increasing amounts of scFv_15_hLi7(■), scFv_15_hLi7m (◆) or scFv_5_hLi7m (●). Immunocomplexes were revealed using peroxidase-conjugated PpL. (**C**) Indirect ELISA: immobilized *L. intermedia* venom and added increasing amounts of scFv_15_hLi7 (■), scFv_15_hLi7m (◆) or scFv_5_hLi7m (●). Immunocomplexes were revealed using peroxidase-conjugated PpL. (**D**) Inhibition of hemolytic activity. Human erythrocytes were incubated with *L. intermedia* venom (0.75 µg) in the presence of different amounts of antibody: 50 pmol (black), 25 pmol (dark grey) and 12.5 pmol (light grey) for 24 h under gentle agitation at 37 °C. Ringer buffer was used as a negative control. The results are expressed in percentage of hemolysis inhibition and the venom alone in the absence of antibody was considered as 100% of hemolysis. Visual inspection of samples is represented in relation to respective antibody fragments. (**E**) in vitro hemolytic assay. Human erythrocytes were incubated with *L. intermedia* venom (10 µg) in the presence of different concentrations of antibody, with (grey) or without (black) normal human serum. Irrelevant scFv αDHEA (200 pmol) and horse hyperimmune serum (SALOX, 1:500) were used as controls. The results are expressed in percentage of hemolysis and the venom alone, in the absence of antibody, was considered as 100% of hemolysis.

The preservation of the recognition profile and specificity of the scFv_15_hLi7m and scFv_5_hLi7m fragments against venom components was confirmed after western blotting. When both fragments were assessed against the venom of different *Loxosceles* species, only the *L. intermedia* venom led to a recognition profile of proteins in the 32–35 kDa molar range, suggesting that the epitope recognized by this fragment is shared by SMaseD isoforms only pertaining to *L. intermedia* (Figure 7A). The ELISA test was used to compare the affinity of the purified scFv_15_hLi7, scFv_15_hLi7m and scFv_5_hLi7m against a recombinant SMase D and the *L. intermedia* whole venom. No improvement in affinity was observed for the mutated version when tested against recombinant LiD1, one of the phospholipases present in the Loxtox family (Figure 7B). However, scFv_5_hLi7m was able to recognize the *L. intermedia* whole venom with greater apparent affinity, thus being expected to produce steric hindrance within the different targets present in the venom (Figure 7C).

### 2.7. L. intermedia Venom Neutralization

In order to assess the humanized antibody fragments capacity to inhibit the hemolytic effects of the *L. intermedia* venom we performed a hemolysis assay, in presence or absence of the complement system. Different amounts of humanized antibody fragments were incubated with human erythrocytes in the presence of *L. intermedia* venom (0.75 and 10 µg mL^−1^). scFv_5_hLi7m (in all molarities) was able to inhibit 100% of the venom’s (0.75 µg mL^−1^) hemolytic activity, when compared to the humanized variants scFv_15_hLi7, the scFv_15_hLi7m or the irrelevant scFv (Figure 7D).

The hemolysis of red blood cells challenged with the venom (10 µg) was strongly inhibited (>90%) in the presence of 50–200 pmol scFv_5_hLi7m, when the complement system was absent (Figure 7E). When normal human serum was added to erythrocytes previously incubated with venom and scFv_5_hLi7m, an inhibition of up to 68% of complement-dependent hemolysis was observed (Figure 7E). Horse polyclonal sera anti-*L. intermedia* venom (SALOX, 1:500) was used as a positive control for hemolysis inhibition under these assay conditions. Additionally, when tested in the presence of 0.75 µg·mL^−1^ and 10 µg·mL^−1^ of *L. intermedia* venom, Limab7 (50 pmol) was able to neutralize 100 and 80% of the hemolysis, respectively (data not shown). An irrelevant scFv anti-DHEA was also employed as a negative control [30].

## 3. Discussion

Today, there is no consensus concerning the efficacy of any reported therapy for the treatment of loxoscelism. It is well-established that SMases D play a key role in dermonecrosis and hemolysis, and a number of studies are focusing on the selection of chemical compounds capable of interfering with SMases activity [31]. In this context, the development of neutralizing therapeutic antibodies directed against SMases may also provide an efficient alternative to conventional serum therapy as currently implemented in Brazil. It has been widely reported that many *Loxosceles* spp. venom proteins induce the production of non-neutralizing antibodies, later present in the total IgG pool of anti-venoms [32,33]. This brings upon the need for the employment of SMases D or their immunorelevant domains as major immunogens in the immunization process, given the significance of these toxins in the course of envenomation [32,34,35,36,37]. In addition to this, over the last years, there has been a rising demand for advancements in the conception of anti-venom production. Despite intensive research to develop alternatives for conventional anti-loxoscelism serum therapy, to date, the LimAb7 still remains the only monoclonal antibody able to neutralize the dermonecrotic activity of the *L. intermedia* venom, as reported by studies in animal models [20]. Recently, we have shown that recombinant LimAb7 antibody fragments (diabodies) preserved in vitro neutralizing capacity of inhibiting SMase D activity, as well as hemolysis induced by *L. intermedia* venom [22]. However, clinical trials of these molecules in humans is not feasible for safety reasons, unless the antibody’s V-domains are humanized. Hence, we designed humanized V-domains and produced them in the scFv format, which is the minimal antibody building block that preserves antigen-binding activity while being well tolerated when administered to humans. We also successfully engineered a PpL binding motif in order to make the purification of antibody fragments using affinity chromatography possible, without requiring the insertion of an epitope tag. This strategy is essential considering future pharmaceutical developments [38,39,40]. The monomeric scFv was produced in bacteria and characterized from a physico-chemical point of view. Our functional studies indicated that the humanized scFv preserved its specificity and antigen-binding activity but did not conserve its capacity of neutralizing SMase D activity in vitro. A slight shift in the interaction surface between the targeted toxin and the antibody upon humanization was suspected by antigen/antibody docking analysis. Overall, these apparently disappointing observations underlined the need to fully understand the mechanism of toxin neutralization prior to the design of an antibody fragment with appropriate format for neutralization.

Antibody humanization demands careful study of a molecule’s structural and conformational features. The most commonly employed method for humanization consists in grafting murine CDRs into whole human framework regions. Still, it has been demonstrated that some murine framework residues, denoted as vernier zone residues, are able to interfere in the CDR loops conformation thus affecting antibody binding affinity [38,41]. Although many methods have been reported for humanization, the immunogenicity of therapeutic antibodies is still a discussed matter [42,43]. The generated antibody fragment must maintain, not only target specificity and high affinity, but also structural and thermal stability as well as preserving or acquiring the capacity to bind to PpL. Considering this, we have produced a minimal size antigen-binding molecule (scFv_15_hLi7) in order to test the intrinsic properties of its humanized V-domains without the influence of additional constant domains. The humanization process did not lead to molecules producing aggregates and no precipitation or degradation phenomena were detected after SEC-HPLC. This is a positive point, taken that the presence of aggregates in drugs of protein nature can cause adverse effects such as reduced drug efficacy, infusion reactions and potentially hypersensitivity reactions [44,45,46].

The scFv_15_hLi7 exhibited a significantly improved humanness with a Z-score in the range of 0. It preserved the recognition specificity of the parental antibody LimAb7, even though its affinity significantly decreased when compared to its murine counterpart according to SPR analysis which were carried out under non-optimal conditions. Indeed, the density of LiD1 protein immobilized on the sensorchip was higher than the optimal value for affinity assessment either because of partial misfolding of LiD1 or random covalent immobilization hiding the epitope surface. We anticipate to better evaluate these parameters as additional data is generated using alternative methods which allow the determination of affinity in free solution [47]. Finally, the recombinant antibody showed a limit of detection of 78 μg·mL^−1^ in ELISA and was able to compete with the parental mouse IgG. Still, the humanized LimAb7 V-domains were not able to neutralize the biological activity of the venom or the recombinant sphingomyelinase when expressed in the scFv format. Aiming to better understand this, we have focused on the SMaseD toxins, which play a key role in loxoscelism. These enzymes have been widely studied and characterized regarding their preferential substrates [48,49], structural basis [1,2,50,51], toxicity [5,52], immunogenicity [53,54] and representativeness in the whole venom [55]. SMases D are also designated dermonecrotic toxins as their recombinant forms have shown to reproduce most of the toxic effects observed in loxoscelism, including dermonecrotic lesions and antigenic properties of the venom [5]. Our SmaseD/LimAb7 and SmaseD/scFv_15_hLi7 docking data clearly support the hypothesis that the interaction between the toxin and the antibody fragments does not occur at the catalytic site of the enzyme. This observation is in agreement with previous overlapping peptide scanning analysis, and also screening of peptide phage-display libraries which identified a putative epitope region located far from the catalytic and Mg^2+^-binding sites of the enzyme [28,29,56]. This is not entirely surprising given that, unlike small neurotoxins which are usually recognized by antibodies at their pharmacological site, larger enzymatic toxins such as PLD are neutralized via other effects such as steric hindrance, making this the first ever reported antibody to neutralize animal toxins via this mechanism [57,58,59]. With its typical minimal size, the monovalent scFv_15_hLi7 could not meet this requirement. The lack of neutralizing ability observed with the scFv fragments (first scFv_15_hLi7 and later with scFv_15_hLi7m) supports the hypothesis of a steric hindrance mechanism for neutralization. Even more important, the results obtained with the murine IgG or its diabody fragment illustrate the difficulty of choosing the most suitable format in terms of size and valence.

The slight shift in the interaction region with the Smase D observed upon the humanization of LimAb7 V-domains may also have significant consequences regarding the affinity of the antibody fragment for the targeted toxin and also its stability. The decrease in the affinity for the toxin and potentially the non-optimal thermal stability we observed for scFv_15_hLi7 could be related to some of the mutations that also altered the VH/VL packing angle. Recent studies have shown that a high degree of cooperation between the VH/VL is required for mutual stabilization and also that a limited number of residues buried inside the antibody domain are critical to maintain the topography of the antigen-binding site [25,39,60]. The mutation H80 A > R slightly impacts the canonical H2 conformation. A charged residue at this position, close to H58 P (CDR H2) and H30 F (CDR H1), could have led to the disruption of an optimal packing of the CDRs and alter the interaction with the target. Hence, residues H103 F > Y; L46 Q > P were back mutated in the revised sequences scFv_15_hLi7m and scFv_5_hLi7m, in an attempt to restore some of the parental antibody’s binding features. As expected, we observed an increased apparent affinity of the second generation scFv_5_hLi7m for the immobilized whole venom (ELISA) as compared with scFv_15_hLi7. However, this was not the case for the recombinant Smase D LiD1 given that this specific toxin may not be the best representative of the *Loxosceles* (Loxtox) phospholipase D family targeted by LimAb7. Indeed, the Loxtox family has been extensively characterized and is comprised of a great amount of Smase D isoforms [6], with distinct biological activities (e.g., hemolytic and sphingomyelinase activities were not significant for LiD1, despite producing dermonecrosis in vivo) [53]. We also observed that the thermal stability of the molecule was not optimal and this may be a consequence of some of the mutations introduced. However, the back mutation of residues H103 and L46, expected to restore the packing angle, did not have any positive impact on the thermal stability. This makes unclear how important it is to preserve the packing angle at least in this particular case study.

The experimental validation of our in-silico docking analysis and humanized fragment re-design was carried out through in vitro hemolysis assays in the presence of the venom and the humanized variants. This approach was selected as a primary screening because it is elucidative and also easier to implement rather than measuring the inhibition of SMase activity in vitro which requires indirect methods with several enzymatic steps sensitive to pH and other potential irrelevant enzyme inhibitors. The crucial role of hemolysis in the context of *Loxosceles* spp. envenoming has been thoroughly described, both via direct and complement dependent activation [52,61,62,63]. The fact that only the diabody neutralizes the hemolysis validates the docking predictions and confirms that neutralization occurs via a steric hindrance mechanism, and corroborates well with previous experiments carried out independently using other experimental strategies [56].

Our findings report for the first time the successful production of a humanized antibody fragment able to neutralize *L. intermedia* venom hemolytic activity in vitro, in a complement dependent (68% of neutralization) and independent manner (100% of neutralization). Therefore, the humanized diabody (scFv_5_Li7m) paves the way for the development of therapeutic recombinant antibodies in loxoscelism. Major advantages of this approach can be foreseen. Immunization of big warm blood animals would no longer be required for production of antivenoms. Additionally, antibody fragments would be prepared without requiring any enzymatic fragmentation that is often deleterious in terms of antigen-binding activity. The specific activity would be higher than the one of polyclonal antivenoms that contain many irrelevant antibodies. Finally, the cDNA encoding the recombinant antibody fragment would be available indefinitely while polyclonal antibodies are never rigorously defined and vary from batch to batch. Of course, diabodies may not be appropriate for human injection due to short half-life and potentially low thermal stability [64]. However, these molecules can be re-designed into Fab fragments which format has yet been proven to be more stable, suitable for human infusion and efficient for the treatment of several diseases [65]. In a Fab, both VH/VL and CH/CL interactions contribute to the functional stability of the antibody fragment and potentially to the in vivo toxin neutralization capacity as suggested previously [66]. In addition, one can consider site-specific PEGylation of scFvs for enhancing the pharmacokinetic properties, the conformational stability, protection from proteolysis and immunogenicity [67]. Altogether, these findings represent a proof of concept on humanized mouse antibodies specific to animal toxins, encouraging the optimized production of molecules for in vivo assessment.

## 4. Materials and Methods

### 4.1. Venoms and Toxins

Vacuum dried venoms from *L. laeta, L. gaucho*, and *L. intermedia* spiders were provided by Centro de Produção e Pesquisa de Imunobiológicos (CPPI, Piraquara, Brazil) and resuspended in a 10 mM Na_2_HPO_4_ buffer containing 137 mM NaCl and 2.7 mM KCl (PBS), pH 7.4 at a concentration of 0.8–1 mg·mL^−1^. The recombinant SMase D LiD1 from *L. intermedia* (Uniprot: P0CE81), presenting 99.67% of identity with LiRecDT1 (PDB: 3RLH) was a gift from Dr. Felicori Figueredo (UFMG, Belo Horizonte, Brazil) (Genbank accession number: AY340702).

### 4.2. Monoclonal and Polyclonal Antibodies

The LimAb7 hybridoma was produced after the immunization of adult female BALB/c mice with *L. intermedia* venom. It has been previously shown to secrete a well-characterized monoclonal IgG_1k_ that neutralizes the democratic activity of *L. intermedia* spider venom, and binds to the SMase D LiD1 as well as several other 32–35kD related proteins of the *L. intermedia* venom, interestingly, not cross-reacting with any components of *L. laeta* and *L. gaucho* venoms, given their considerable interspecies homology [20,56]. Horse hyperimune sera (SALOX) reactive to *L. intermedia*, *L. laeta*, and *L. gaucho* venoms was produced by CPPI. Horse IgG F(ab)’_2_ anti-*L. intermedia* venom was prepared as previously reported [20].

### 4.3. Bacteria

The *Escherichia coli* AD494 (DE3) pLysS strain (Novae, Nottingham, UK) and HB2151 (Stratagene, La Jolla, CA, USA) were selected for protein expression. Bacteria culture media was purchased from AthenaES (Baltimore, MD, USA) and all chemicals were of standard grade and acquired from Sigma Aldrich (St. Louis, MO, USA) or equivalent.

### 4.4. Protein Quantification

Protein concentration was determined by the Bradford reagent (Bio-Rad Laboratories, Hercules, CA, USA). Alternatively, purified protein concentration was measured by the absorbance at 280 nm using, for each purified protein, the molar extinction coefficients (ε) determined as previously reported [68].

### 4.5. Humanization of Antibody V-Domains

The structure of mouse LimAb7 Fv (Genbank accession number KT381972) was previously modeled [69]. Amino acid numbering and sequence analysis were carried out using the Web interface IMGT tools and database (IMGT/DomainGapAlign). Humanized versions of LimAb7 V-domains were generated by grafting all six CDRs onto human IGKV and IGHV domains having high sequence identity and closely related canonical classes with LimAb7 V-domains. The protocol was adapted from a previous one [24]. After grafting the CDRs to human FR regions, each amino acid (AA) substitution was inspected individually, based on the physico-chemical classes of the AA differences [70]. We considered the humanness score (Z-score), which compares the sequences with a set of known human sequences assigned to germline derived families, aiming to achieve a score close or above 0 [71]. VH/VL packing angle and residues that might play an important role in maintaining the correct binding-site topography were identified [25]. Additional refinements were carried out in IGKV-FR1 in order to generate a PpL-binding site without altering predicted humanization and residual immunogenicity as previously suggested [27,72]. Finally, the designed sequences were compared with human germline genes in order to calculate a human germinality [73].

### 4.6. Generation and Purification of Humanized scFv_15_hLi7 and scFv_5_hLi7m

Codon optimized DNA encoding humanized V-domains fused together in the VH–VL orientation via a sequence encoding the (G_4_S)_3_ linker were synthesized at Genscript (Piscataway, NJ, USA) and cloned into the prokaryotic expression vectors pET-22b (+) or pSW1, in frame with the PelB signal sequence [74].

Production and PpL-purification of scFv_15_hLi7 (first generation of the humanized scFv) were carried out under generic conditions at Genscript. Purified scFv_15_hLi7 concentration was adjusted to 0.57 mg·mL^−1^ in PBS pH 7.2. Aliquoted samples were stored at −80 °C.

For the scFv_15_hLi7m’s (second generation of the humanized scFv) construction, two *Bam*HI restriction sites were introduced in the nucleotide sequence encoding the (G_4_S)_3_ linker. Therefore, double digestion with *Bam*HI followed by self-ligation of the plasmid allowed the generation of a vector encoding diabody scFv_5_hLi7m which differs from scFv_15_hLi7m by the size of its linker peptide (G_4_S). Both scFvs were produced by *E. coli* AD494 (DE3) pLysS bacteria transformed with vector pET-22b (+) containing the insert of interest as previously described [22]. Alternatively, we used HB2151 strains transformed with pSW1 vector for scFv production under generic conditions and PpL capture [75]. The scFv-containing fractions were pooled and subjected to dialysis in PBS, pH 7.4 overnight at 4 °C.

### 4.7. SDS-PAGE, Western Blot and Dot Blot

To assess bacterial periplasmic extracts for scFv expression, samples were resolved by SDS-PAGE under non-reducing conditions on 12.5% polyacrylamide gel. Proteins migrated at 150 V for 2 h at room temperature. Subsequently, gels were either stained with Coomassie Brilliant Blue for protein identification or transferred onto a 0.45 mm nitrocellulose membrane, for 2 h, 100 V at 4 °C. The quality of the transfer was checked by transitional staining with Red Ponceau. In order to verify the expression of the scFvs, the membrane’s non-specific binding sites were first blocked for 1 h in PBS containing 5% (w/v) non-fat dry milk and 0.3% (v/v) Tween 20. Next, membranes were incubated with peroxidase-conjugated PpL (ThermoFisher, Waltham, MA, USA) in PBS, pH 7.4, containing 0.05% Tween 20, for 1 h at 37 °C and stained with DAB/chloronaphtol.

Aiming to confirm the fragments specificity against the *L. intermedia* venom, the periplasmic extracts were analysed by dot blot. Firstly, *L. laeta*, *L. gaucho*, and *L. intermedia* venom (2.5 μg each) were immobilized on to nitrocellulose membrane, then the non-specific binding sites were blocked with non-fat milk as described above. Next, membranes were incubated with the scFv periplasmic extracts (for 1 h at 37 °C) and after with peroxidase-conjugated PpL (ThermoFisher) for scFv detection. Lastly, membranes were stained with DAB/chloronaphtol.

Additionally, *L. laeta*, *L. gaucho*, *L. intermedia* venoms, and recombinant Smase D LiD1 were subjected to an SDS-PAGE on a 15% acrylamide gel and transferred onto a 0.45 mm nitrocellulose membrane in order to verify the scFv’s binding to the dermonecrotic toxins. The immunocomplexes were detected with peroxidase-conjugated PpL (ThermoFisher) or peroxidase-conjugated rabbit anti-mouse IgG (Sigma Aldrich, 1:4000). Between all intermediate steps, three washings with PBS (pH 7.4) containing 0.05% Tween 20 were performed.

### 4.8. UV-Vis Analysis

UV-Vis spectra (from 220 to 350 nm) were carried out in triplicate after dilution of samples at the 0.5 AU_280 nm_ in PBS pH 7.2.

### 4.9. Size-Exclusion Chromatography

100 µL of PpL-purified scFv (2 or 10 μM) were analysed by size-exclusion high pressure liquid chromatography (SEC-HPLC) using a prepacked Superdex 75 10/300 GL column calibrated with standards from GE Healthcare (Buc, France). Proteins were eluted with PBS pH 7.2 at a rate of 0.5 mL·min^−1^ and detected with a UV recorder at 280 nm.

### 4.10. Circular Dichroism

CD spectra were obtained on a Model CD6 spectrometer (Jobin-Yvon-Spex, Longjumeau, France) at different temperatures from 15 to 60 °C using a thermoregulated bath using a quartz sample cell with a 1 mm path length. The ellipticity was scanned from 200 to 250 nm with an increment of 1 nm, an integration time of 2 s, and a constant band-pass of 2 nm. The concentration of the scFv_15_hLi7 protein was adjusted to 0.114 mg·mL^−1^ in PBS, pH 7.4.

### 4.11. Nano-DSF

The samples (10 μM) were loaded in standard nano-DSF capillaries and measured using the Prometheus NT.48 instrument (NanoTemper, Munich, Germany) containing aggregation optics. The LED intensity was set to 10%, whereas the temperature ramp was set from 20 to 95 °C with 1 °C per min. As negative control one duplicate was integrated containing heat denatured protein.

### 4.12. Enzyme-Linked Immunosorbent Assay

Sandwich ELISA: Plates (Nunc MaxiSorp™, ThermoFisher) were coated with 100 μL of a 10 μg·mL^−1^ solution of IgG F(ab)’_2_ horse anti- *L. intermedia* venom in carbonate buffer pH 9.6 at 4 °C overnight. After blocking (2% casein in PBS), 100 µL of *L. intermedia* venom (0.039–10 µg·mL^−1^) were added and incubated for 1 h at 37 °C. The plates were washed and incubated with solution of scFv_15_hLi7 (20 μg·mL^−1^). Lastly, 100 μL of peroxidase-conjugated PpL (ThermoFisher) were added for 1 h at room temperature.

Competitive ELISA: Plates were coated with *L. intermedia* venom (100 µL, 10 µg mL^−1^) for 16 h at 4 °C and then saturated with 2% casein diluted in PBS for 60 min, at 37 °C. Next, solutions containing LimAb7 (1 µg·mL ^−1^) and scFv_15_hLi7 (6.25–100 µg·mL^−1^) were incubated for 1 h, at 37 °C. Immunocomplexes formed with the IgG were detected by adding peroxidase-conjugated anti-mouse IgG (1:4.000, Sigma, St. Louis, MO, USA).

Indirect ELISA: Plates were coated with *L. intermedia* venom or SMase D Lid1 (100 µL, 2.5 µg·mL^−1^) for 16 h at 4 °C, and then saturated with 2% casein diluted in PBS for 60 min. Further, different concentrations of scFv (0.156–20 µg·mL^−1^) were added at 37 °C for 1 h. Immunocomplexes were detected by adding peroxidase-conjugated PpL (ThermoFisher).

In both ELISA formats, immunocomplexes were revealed by the addition of substrate (0.2% 2,2/-6azino-bis (2-ethylbenzthiazoline-6-sulphonic acid) to a 0.05 M citric acid buffer, pH 4.0 containing 0.015% hydrogen peroxide (Sigma, St. Louis, MO, USA), for 15 min. Absorbance at 405 nm was measured using an ELISA plate reader. All incubation steps were carried out at 37 °C. All assays were conducted in triplicates. ELISA standard curves were fitted by non-linear regression, using the Saturation Binding - One site -Specific binding function. Statistical analyses and graphics were performed in GraphPad Prism v7.0 for MacOSX (GraphPad Software, San Diego, CA, USA).

### 4.13. SPR Analysis

The BIAcore T100 instrument and all reagents were obtained from GE Healthcare Life Sciences, SMase D LiD1 was covalently attached to a CM5-sensor chip using standard amine coupling through EDC/NHS chemistry (approximately 3500 RU). The purified IgG and scFv diluted in PBS, pH 7.4, were passed over the immobilized target (LiD1) at a flow rate of 30 µL·min^−1^ for 1 min at 25 °C. The binding kinetics were analyzed using the single-cycle kinetic method with no regeneration between sample injections. Experimental Rmax were 8750 RU and 2900 RU for the IgG and the scFv, respectively. Kinetic constants (k_a_, k_d_) were deduced from the analysis of association and dissociation rates of at least four different antibody concentrations (0.125 µM–2.0 µM). The dissociation constant K_D_ was calculated as K_D_ = k_a_/k_d_. Sensorgrams were analysed using the BIA evaluation version 2.0.2 software (GE Healthcare, Vélizy-Villacoublay, France). All experiments were carried out in duplicate.

### 4.14. Modelling and Molecular Docking

In order to investigate which amino acid residues might participate in the antigen-antibody interaction, we performed homology modelling of murine monoclonal antibody LimAb7 and scFv_15_hLi7 using the MODELLER tool (version 9.19, UCSF—University of California, San Francisco (UCSF), San Francisco, CA, USA) [76]. In this step, antibody sequences were aligned through a BLAST [77] search and their templates were identified based on sequence identity, query coverage and E-value criteria (Table S2). Utilizing the template choices for each antibody, 1000 models were generated with the MODELLER tool (9.2v) and then selected according to their ZDOPE score values [78]. Subsequently, model quality was evaluated by comparing the predicted structures with their respective templates via superimposition and atomic RMSD (root mean square deviation) assessment. The chosen cu-off RMSD values of Cα trace between all homology structures and templates was <2.00 Å. Moreover, model energy minimization and loop refinement were carried out in attempt to increase the model’s quality (Ramachandran plot) using the Chimera software [79] and MODELLER tool, ensuring more than 90% of models’ AA residues were in favored Ramachandran Plot regions. After modelling the three structures, the ClusPro2.0 server (Boston University (BU), Boston, MA, USA) [80] was used to predict the interactions between the modelled antibodies and the Smase D LiRecDT1 (PDB: 3RLH). The LiRecDT1 SMase was selected instead of SMase LiD1 given its X-ray data is available and it only differs from LiD1 by two AA residues. The antibody mode was selected with the non-CDR regions masked automatically [81]. The antibody structures were submitted as the receptor and SMase D LiRecDT1 as the ligand. ClusPro selected the 1000 best scoring solutions, clustered them according to RMSD criteria, and the lowest ClusPro energy score, representing the greatest probability of antigen-antibody interaction, was selected [80]. Amino acid contacts and interaction parameters between the antibodies and their targets were assessed through the PDBSum online platform [82].

### 4.15. Neutralization of the Hemolytic Activity

Blood from human healthy donors was collected in tubes containing sodium citrate buffer (BD Plastipak, Franklin Lakes, NJ USA). The platelet-rich plasma and buffy coat were removed by aspiration after centrifugation at 200× *g* for 15 min. Packed erythrocytes were washed three times with Ringer Solution (125 mM NaCl, 5 mM KCl, 1 mM MgSO_4_, 32 mM HEPES, 5 mM glucose, 1 mM CaCl_2_, pH 7.4, 300 mOsm/kg H_2_O) and redissolved at a final concentration of 10^8^ cells·mL^−1^. In order to evaluate the neutralization potential of the antibodies, 0.75 μg of venom were incubated with the following antibody molarities (12.5–50 pmol). After 24 h of gentle agitation and incubation at 37 °C, samples were centrifuged (5 min, 200× *g*) and the absorbance at 570 nm of the supernatants was read. Samples were analyzed in triplicates along with negative (Ringer solution) and positive (distilled water containing 0.1% (v/v) Triton X-100) controls. Absorbance values were converted to percentage of hemolysis considering the absorbance at 570 nm of 0.75 μg of venom as 100% lysis.

Subsequently, the inhibition of hemolysis was evaluated in the presence of the components of the complement system. In order to do this, a 1 mL solution containing 10^8^ erythrocytes was treated with *L. intermedia* venom (10 µg) in the presence of different antibody quantities (25–200 pmol) for 24 h under gentle agitation and incubation at 37 °C. Samples were centrifuged (5 min, 200× *g*) and the absorbance at 570 nm of the supernatants was read. Next, erythrocytes were washed three times with Ringer solution and incubated with 500 µL of a solution of normal human serum, diluted 1:2, for one hour at 37 °C. Unlysed cells were centrifuged and the absorbance of the supernatant was measured at 570 nm and expressed as percentage of hemolysis. After subtracting all samples absorbance from the absorbance at 570 nm obtained from Ringer treated erytrocytes, before and after complement incubation, the lysis percentage (absorbance _sample_ / (absorbance _venom without / complement_ + absorbance _venom with / complement)_) × 100 was calculated considering the sum of the absorbance at 570 nm of the venom before complement incubation and absorbance at 570 nm of venom after complement incubation as 100% of hemolysis. Mean and standard deviation were determined from duplicate samples. 

This study has been approved by the human research ethics committee from Setor de Ciências da Saúde do Universidade Federal do Paraná (Curitiba, Brazil) under the certificate number CEP/SD2911004, CAAE:93959218.7.0000.0102 on 24 September 2018.

## Supplementary Materials

The following are available online at https://www.mdpi.com/2072-6651/12/4/256/s1, Table S1: Hydrogen Bonds between the amino acid residues of the antibodies and Lid1, Table S2: In silico modeling data.
