# Supplementary material for: Loxoscelism: Advances and Challenges in the Design of Antibody Fragments with Therapeutic Potential
Source: Toxins (Basel). 2020 Apr 16;12(4):256. doi: 10.3390/toxins12040256 (PMC7232456; doi:10.3390/toxins12040256)
Supplement: Supplementary file 1 [file toxins-12-00256-s001.pdf]

Supplementary Materials: Loxoscelism: Advances and Challenges in the Design of Antibody Fragments with Therapeutic Potential

Sabrina Karim-Silva, Alessandra Becker-Finco, Isabella Gizzi Jiacomini, Fanny Boursin, Arnaud Leroy, Magali Noiray, Juliana de Moura, Nicolas Aubrey, Philippe Billiald * and Larissa M. Alvarenga *

Table S1. Hydrogen Bonds between the amino acid residues of the antibodies and Lid1.

Antibody	Antibody Domain	Amino Acids		Described Hotspots of Relevance in Ag	Distances (Å)
		Antibody(Ab)	Antigen (Ag)		
**LimAb7**	H:CDR1	Ser31	Lys59	Felicori et al; 2006	2.68
	H:CDR2	Tyr52	Asn56	Murakami et al; 2005	2.87
	H:CDR2	Tyr52	Lys58	Felicori et al; 2006	2.84
	H:CDR2	Asp55	Lys58	Felicori et al; 2006	2.41
	H:CDR3	Tyr103	Gly18		2.73
	H:CDR3	Tyr103	Lys59		2.69
	H:CDR3	Tyr106	Arg55	Murakami et al; 2005	3.03
	H:CDR3	Tyr107	Gly54	Murakami et al; 2005	2.74
	L:CDR2	Val33	Lys234	De Moura et al; 2011	2.66
	L:CDR2	Asn34	Arg235	De Moura et al; 2011	3.29
	L:CDR2	Asn34	Asp233	De Moura et al; 2011	3.01
	L:CDR2	Glu35	Arg235		3.02
	L:CDR2	Glu35	Ser236		3.00
	L:CDR2	Asn36	Arg235		2.61
**scFv$_{15}$hLi7**	H:CDR1	Ser31	Lys59	Felicori et al; 2006	2.58
	H:CDR1	Asp55	Asn56	Murakami et al; 2005	3.08
	H:CDR2	Asp55	Lys58	Felicori et al; 2006	2.77
	H:CDR2	Tyr101	Arg55		2.84
	H:CDR2	Tyr103	Asp21		2.91
	H:CDR2	Lys105	Asp21	Felicori et al; 2009	2.72
	H:CDR2	Lys105	Asp25	Felicori et al; 2009	2.56
	H:CDR2	Tyr106	Glu22		2.91
	H:CDR2	Tyr106	Arg55	Murakami et al; 2005	2.79
	H:CDR3	Tyr107	Asp255		2.85
	H:CDR3	Tyr108	Asp255		2.81
	L:CDR2	Val33	Lys234	De Moura et al; 2011	2.74
	L:CDR2	Asn34	Arg235	De Moura et al; 2011	2.92
	L:CDR2	Glu35	Arg235		2.85
	L:CDR2	Glu35	Arg235		3.02
	L:CDR2	Glu35	Ser236		2.95
	L:CDR2	Asn36	Arg235		2.87
	L:CDR2	Asn36	Arg235		2.90
	L:CDR2	Asn36	Asp255		2.77
	L:CDR2	Asn37	Arg235		2.74

Table S2. In silico modelling data.

Antibodies	Template (PDB:chain)	% Identity	Resolution (Å)	RMSD (Å)
LimAb7	5YD3_A	59%	1.35	0.871
scFv$_{15}$hLi7	5YD3_A	61.5%	1.35	0.659
